# Evidence for discrete solar and lunar orientation mechanisms in the beach amphipod, *Talitrus saltator* Montagu (Crustacea, Amphipoda)

**DOI:** 10.1038/srep35575

**Published:** 2016-10-19

**Authors:** Alberto Ugolini, Laura S. Hoelters, Alice Ciofini, Vittorio Pasquali, David C. Wilcockson

**Affiliations:** 1 Università di Firenze, Dipartimento di Biologia, Via Romana 17, 50125 Firenze, Italy; 2 Institute of Biological Environmental and Rural Sciences, Aberystwyth University, Penglais, Aberystwyth, SY23 3DA, UK; 3 Dipartimento di Psicologia, Università di Roma “La Sapienza”, Via dei Marsi 78, 00185 Roma, Italy

## Abstract

Animals that use astronomical cues to orientate must make continuous adjustment to account for temporal changes in azimuth caused by Earth’s rotation. For example, the Monarch butterfly possesses a time-compensated sun compass dependent upon a circadian clock in the antennae. The amphipod *Talitrus saltator* possesses both a sun compass and a moon compass. We reasoned that the time-compensated compass mechanism that enables solar orientation of *T. saltator* is located in the antennae, as is the case for Monarch butterflies. We examined activity rhythms and orientation of sandhoppers with antennae surgically removed, or unilaterally occluded with black paint. Removing or painting the antennae did not affect daily activity rhythms or competence to orientate using the sun. However, when tested at night these animals were unable to orientate correctly to the moon. We subsequently measured circadian gene expression in the antennae and brain of *T. saltator* and show the clock genes *period* and *cryptochrome 2* are rhythmically expressed in both tissues, reminiscent of other arthropods known to possess antennal clocks. Together, our behavioural and molecular data suggest that, *T. saltator* has anatomically discrete lunar and solar orientation apparatus; a sun compass, likely located in the brain and a moon compass in the antennae.

Many organisms use visible objects in their natural environment, including celestial bodies, as reference points to orientate appropriately. However, because of Earth’s rotation celestial objects appear to move across the sky over the course of a 24 h daily cycle and so orienting using these cues necessitates continuous adjustment to maintain a constant direction. A classic model of this, the Monarch butterfly *Danaus plexippus*, uses the sun as an orientation cue during its remarkable annual migrations but to maintain a constant flight direction, compensates for azimuthal variation of the sun by means of a circadian timing mechanism^1^. Remarkably, in *D. plexippus*, in addition to the circadian clock found in the central brain, an extra-cerebral clock localised in the antennae is essential for appropriate time-compensated solar orientation; the removal of antennae, or painting them black unilaterally, disables correct flight orientation^2^,^3^,^4^.

The consensus arthropod model of the molecular circadian clock of fruit flies is centred on transcriptional-translational feedback loops where the positive transcription factors CLOCK (CLK) and CYCLE (CYC) drive the transcription of negative elements *period* and *timeless* the cognate proteins of which accumulate in the cytoplasm, form a heterodimer and translocate to the nucleus to suppress their own expression, a cycle that lasts about 24 h owing to regulatory post-translational events (for detailed reviews see^5^,^6^). Molecular characterisation of the circadian clock in the Monarch brain revealed a novel negative transcriptional feedback loop with CLK and CYC driving rhythmic transcription of *period*, *timeless* and *cryptochrome 2 (cry*2), the protein product of the latter feeding back as the main repressor of CLK:CYC^7^,^8^. Later work determined that similar feedback loops were central to the timing mechanism found in the antennae but entirely separate from the brain clock. Indeed, Monarch antennal clocks can be synchronised, or entrained, to light and dark (L:D) cycles even when physically separated from the head of the animal^3^.

The sandhopper *Talitrus saltator* is one of the best-known biological models for studies on compass mechanisms in littoral arthropods^9^. Since the 1950’s it has been demonstrated that supralittoral sandhoppers artificially or naturally displaced from the band of damp sand where they live during the day use multiple cues in their recovery to their preferred shore zone, following the shortest route (i.e. the sea – land axis of sandy beaches). This is important to minimize the effects of biotic and abiotic stressors present in that environment, such as predation, variations in temperature, salinity and relative humidity^10^. For the same reasons, sandhoppers evolved nocturnal habits. In nature, *T. saltator* emerges from its burrows at night and performs inland excursions to forage, before returning to the belt of damp sand at the water’s edge before sunrise. This rhythm of locomotor activity is endogenous and synchronized to the natural 24 h diurnal cycle by the dawn^11^. However, because sandhoppers can also be displaced from their refuges during the day by natural and artificial disturbances, they must be able to compensate for the azimuthal variation of the celestial bodies over the entire day/night cycle.

Previous studies have shown that the endogenous, circadian clock mechanism, regulating the rhythmic locomotor activity exhibited by *T. saltator*, is also involved in sun compass orientation behaviour^10^. Intriguingly, nocturnal orientation using lunar cues appears to rely on a separate time-compensatory mechanism^12^,^13^. Comparison of the anatomical organisation of Monarch time-compensated navigation and the behavioural orientation of sandhoppers raises the question of whether sandhoppers evolved anatomically discrete, dual systems to enable them to orientate at night-time and daytime to locate their preferred foraging and resting sites. To address this question we performed (a) surgical removal or (b) unilateral occlusion of antennae with black paint and assessed the solar and lunar orientation of animals under natural light regimes in a zeroed horizontal component of the natural magnetic field. This experimental approach enabled us to test whether solar and lunar orientation is mediated by mechanisms in the antennae and to establish whether these are anatomically separate from the timing mechanisms that control daily locomotory activity rhythm. Moreover, we evaluate if the directional choice of antennaless sandhoppers is really based on the sun or moon compass instead of other compass cues (such as the geomagnetic field).

## Materials and Methods

### Animals and husbandry

Adult individuals of *T. saltator* were collected from a sandy beach in the Natural Park of Migliarino, San Rossore, Massaciuccoli, Pisa, Italy (43°40′03″N, 10°20′29″E), seaward direction = 265°, in June–July 2015 and June 2016. Experimental orientation releases were performed no more than 21 days from collection and were made under a clear sky and with an unobstructed view of the sun or moon.

In the laboratory, animals were kept in Plexiglas boxes containing damp sand at ambient temperature (25 °C) and with an artificial photoperiod (L:D = 12:12) in phase with the natural L:D cycle (i.e. with same mid-point for the light phase). We opted to maintain experimental animals on a L:D = 12:12 regime (i.e. 15° per hour of sun azimuthal variation) to avoid any influence of the hours of light on the speed of time-compensation of the azimuthal variation of the sun (see Ugolini *et al.*^14^). Food (universal dried food for fish, SERA Vipan, Heisenberg, Germany) was available *ad libitum*.

### Sun compass experiments

**Figure 1:**
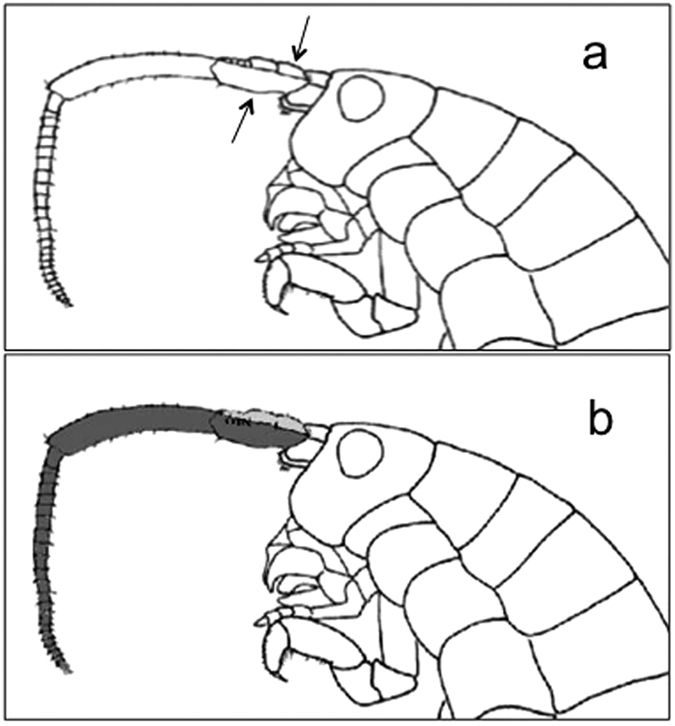
Surgical removal of the antennae. (**a**) Schematic representation of the removal of the first and second antennae (arrows show the approximate position of cutting on first and second antennae), and (**b**) the unilateral painting of the first (light grey) and second (dark grey) antennae. Redrawn from Ruffo^45^.

Individuals were divided into: (1) intact animals, (2) animals with both first and second antennae surgically removed (Fig. 1a), and (3) animals with right antennae painted with black enamel (Fig. 1b). Members of each group were relocated to the laboratory (with an L:D = 12:12 cycle in phase with the natural photoperiod) where activity was monitored using a micro-wave radar system (see Pasquali and Renzi^15^ and Ugolini *et al.*^16^ for a detailed description of the device).

Experimental orientation releases were performed in Florence in June 2016, after at least 10 days of activity monitoring, between 1130 hours and 1300 hours. Taking into account similar experiments carried out on *D. plexippus*^2^, we also painted antennae with black enamel to test if discordant timing between antennae affects the sun or moon compass. Sandhoppers with right antennae painted with black enamel (Fig. 1b, Rainbow, Maimeri S.p.A., Mediglia, Milano, Italy) were tested 24 hrs after painting.

It has previously been demonstrated that testing sandhoppers in small groups does not influence their directional choice^17^. Therefore, for each trial 5–10 individuals were released into a transparent Plexiglas bowl, filled to a depth of about 0.5 cm with seawater. Seawater induces sandhoppers to rapidly orientate toward the landward direction of their home beach. The bowl was placed horizontally on a transparent plate so that the sandhoppers could be observed from below. The bowl and plate were mounted on a tripod and surrounded by a cylindrical screen 1–3 cm high allowing the animals in the bowl only the vision of the sun and sky^18^. In order to carry out releases with a zeroed (or very reduced) horizontal component of the natural magnetic field that could constitute a further orienting reference^19^, we equipped the tripod with a pair of Helmholtz coils (diameter = 64 cm, distance = 35 cm) supplied by a battery and regulated by an electronic rheostat. A single direction for each sandhopper was determined 2 minutes after the introduction of the animals to the bowl. Directions were measured from freeze-frame images recorded by a video camera placed below the bowl or read directly by the experimenter (error ±2.5° in both cases).

### Moon compass experiments

For moon compass experiments, animals were prepared exactly as for sun compass experiments; i.e. individuals were divided into: (1) intact animals, (2) operated without first and second antennae (Fig. 1a), (3) right antennae painted with black enamel (Fig. 1b). Tests were conducted in July 2015 in the same conditions as for sun compass tests, under full moon phase (95% illuminated fraction). The direction of each individual was recorded by infra-red (IR) sensitive video camera placed below the bowl and measured from freeze-frame images, as previously described^13^. The bowl was illuminated with an IR source, placed approximately 2 m from the bowl. Previous studies have shown that *T. saltator* is not sensitive to IR^20^,^21^.

### Clock gene expression analysis

Cerebral ganglia and antennae (first and second) were harvested separately from *T. saltator* after entrainment under 12:12 L:D cycles followed by a 24 h free-running period in DD. All tissues were preserved in RNAlater at −20 °C until extraction. Total RNA was extracted, reverse transcribed and subjected to qPCR using Taqman**™** hydrolysis probes (Thermo Life Sciences, UK) and absolute quantitation according to previously published methods^22^ (see also, Supplementary Methods). Gene sequences for primer and probe design were obtained from the brain transcriptome of *T. saltator*^23^. Data were expressed as copy number of each target gene transcript per copy number of the reference gene *arginine kinase*.

### Statistical analysis

In all the orientation experiments only one direction per individual was recorded. Directional data were analysed using the methods proposed by Batschelet^24^ for circular distributions. For each distribution the length of mean resultant vector and the mean angle were calculated. To assess the non- uniformity of distributions the Rao’s test has been used (P < 0.05 at least).

Rhythmicity in clock gene expression was determined using Cosinor software (http://www.circadian.org/softwar.html) developed by Roberto Refinetti^25^ and using default circadian parameters.

## Results

### Antennae are not required for sun-compass orientation

Initially we reasoned that the time-compensated orientation mechanism of *T. saltator* resides in the antennae, as is the case for Monarch butterflies, and tested the behaviour of animals with antennae surgically removed or unilaterally painted black and under the sun to investigate whether they play a role in the solar orientation.

**Figure 2:**
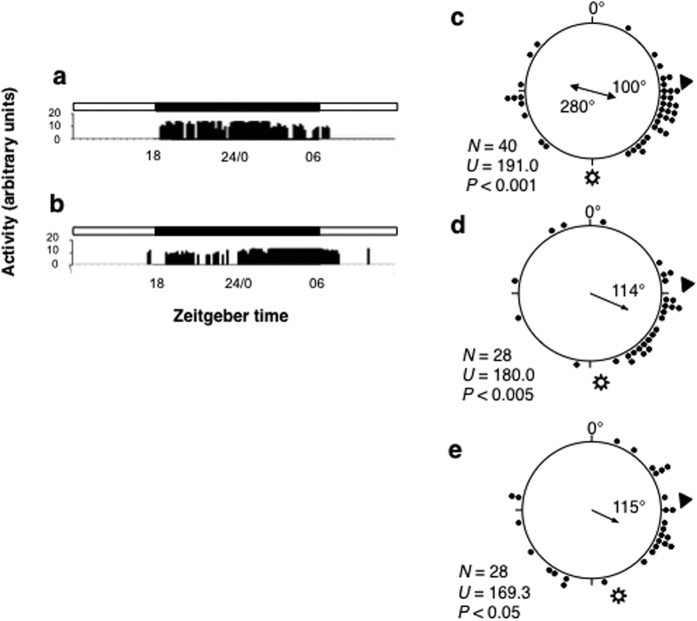
Daily locomotor activity and solar orientation are unaffected by antennal ablation . Locomotor activity of intact (**a**) and antennae ablated animals (**b**) in L:D 12:12. Black and white bars indicate light and dark respectively. The quantity of activity (recorded simultaneously from a group of twenty individuals in each experiment) is expressed in arbitrary units. Distributions obtained testing: intact (**c**), antennaless (**d**) and right antennae black painted (**e**) animals. 0°, North (zeroed magnetic field); arrow inside the distributions: mean vector and angle (length of mean vectors: 0 to 1 = radius of the circle); black dots: sandhoppers’ directions (each dot corresponds to the direction of one individual); black triangles outside the distributions: landward direction of sandhoppers’ home beach. Sun symbol: solar azimuth at the time of the test. *N*, sample size. *U*, Rao’s test values with probability levels (*P*).

Daily activity rhythms of all animals remained unaffected following antennal removal (Fig. 2a,b). Directional choices of in intact individuals showed a clear bimodality in accordance with the Y-axis of their home beach (Fig. 2c) and the mean landward direction of these animals was very similar to those of sandhoppers without antennae (only 14**°** different, Fig. 2d). Moreover, releases of animals with unilateral first and second antennae obscured with black paint also showed a mean direction in close agreement with the landward direction of their home beach (30**°** different from expected, Fig. 2e). Therefore, our results demonstrate that antennae are not essential in the solar orientation of *T. saltator* since their removal or unilateral painting does not disrupt the of sea-land direction finding based on the sun compass (Fig. 2c–e).

### Antennae are required for moon-compass orientation

**Figure 3:**
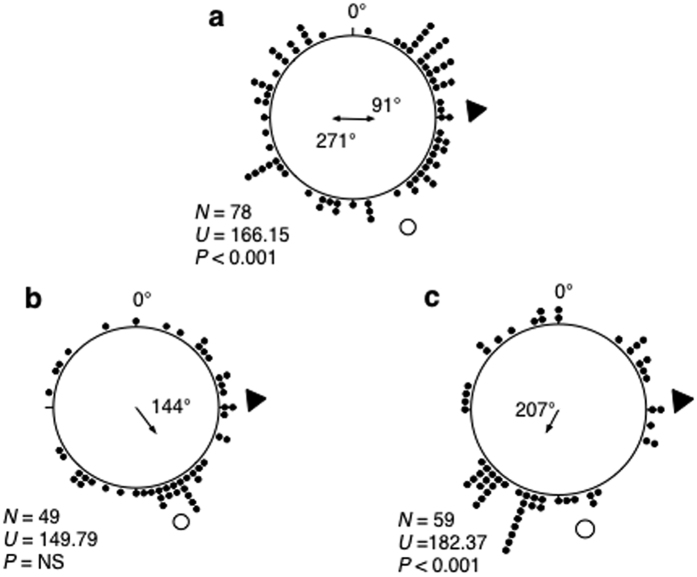
Antennal manipulation disrupts lunar orientation. Distributions obtained testing: intact (**a**), antennaless (**b**) and right antennae painted black (**c**) animals held under L:D12:12 (with lighting in phase with the natural photoperiod). 0°, North (zeroed magnetic field). For further explanations see Fig. 2.

Next we tested lunar orientation of sandhoppers by performing the same experimental releases at night, under a full moon (95% illuminated fraction). Figure 3a clearly shows that whilst the intact individuals exhibit a bimodal distribution in accordance with the expected orientation of animals migrating up or down-shore (i.e. the sea–land axis of their home beach), individuals lacking first and second antennae (Fig. 3b), or with their first and second antennae unilaterally painted black (Fig. 3c), did not orientate in the expected direction (59**°** and 122**°** different, respectively) but instead displayed a photopositive orientation directed toward the source of light (i.e. the moon). Thus, sandhoppers lacking antennae or unilaterally blinded with paint do not lose competence to orientate using the sun as a chronometrically compensated astronomic cue, but are unable to use the moon as a reference in the same way.

### Both the brain and antennae express canonical clock gene transcripts

**Figure 4:**
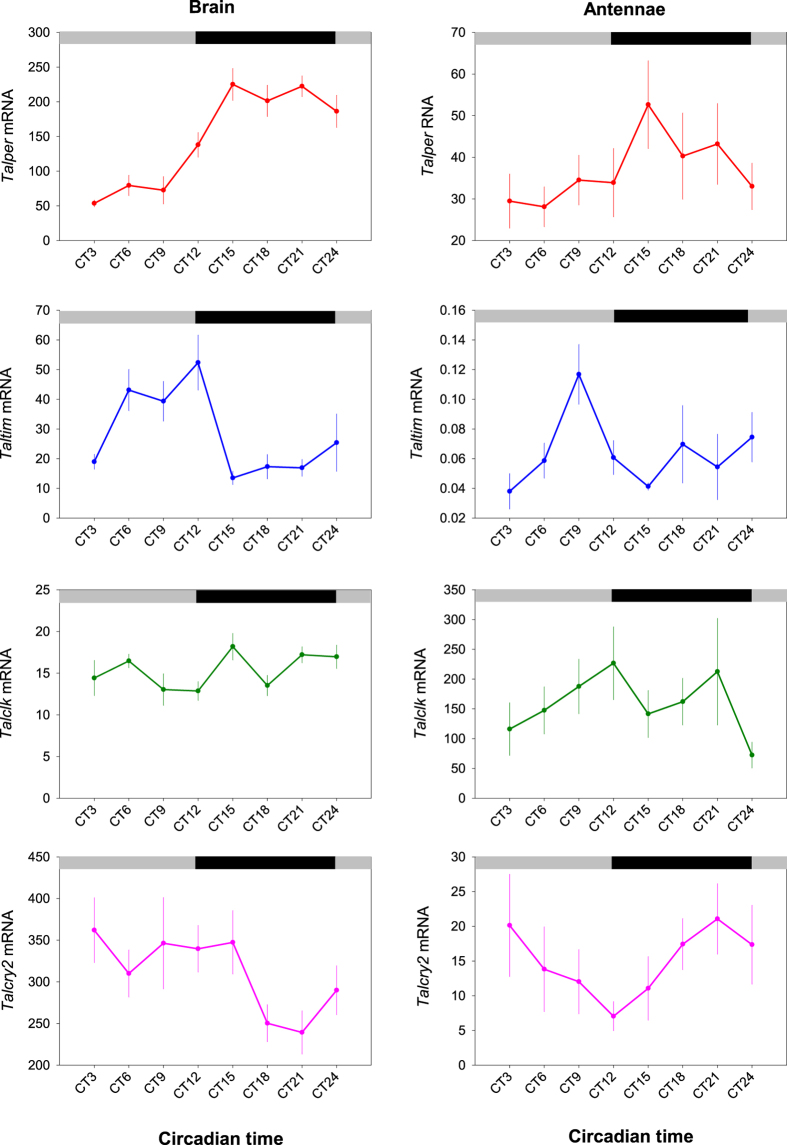
Brain and antennae show rhythmic clock gene expression in constant darkness. Circadian clock gene transcripts were detected in the brain (left panel) and antennae (right panel). Quantification of these transcripts over a 24 h period revealed rhythmic accumulation of *Talper* and *Talcry2* in both tissues by cosinor analysis: Brain, *Talper*, *F* = 17.42, *P* = 0.0067, *Talcry2*, *F* = 18.84, *P* = 0.0056; Antennae, *Talper*, *F* = 6.61, *P* = 0.04, *Talcry2*, *F* = 15.18, *P* = 0.009). *Talclk* and *Taltim* were not rhythmic within a circadian period. Data are expressed as copies RNA per copy of the reference gene arginine kinase. Data are mean +/− sem from 6 biological replicates of 5 pooled heads. Grey and black bars show time of expected light and dark respectively.

The consensus model of the core circadian clock mechanism is based on transcriptional-translational autoregulatory feedback loops^5^, the molecular elements of which are well conserved across diverse taxa^26^,^27^. A feature of this system in many organisms is that core clock transcripts show rhythmic changes in abundance over a 24 h period. Therefore, demonstrating cycling expression of clock genes in the antennae of *T. saltator* in free-running (constant darkness, DD) conditions would support the notion that these appendages have the potential to keep time. We used Taqman™ qPCR assays to measure the temporal expression profiles of the canonical clock gene homologues *period*, *timeless*, *clock*, and *cryptochrome 2* in animals held in DD following entrainment in L:D 12:12 (Fig. 4). Cosinor analysis of gene expression profiles revealed rhythmicity in *Talper* (peak expression between CT15–21) and *Talcry2* (peak expression between CT3–15) in the brain. *Timeless* expression in the brain was not shown to be rhythmic by Cosinor analysis. *Talclk* was not significantly rhythmic. In the antennae only *Talper and Talcry2* cycled (Cosinor), with mRNA abundance peaking at around CT15–18, similar to that of the brain. However, antennal *Talcry2* expression was in antiphase to that in the brain with peak abundance between CT21 and CT3 and low levels at CT9–CT15.

## Discussion

We set out to test whether the solar and lunar orientation mechanisms in *Talitrus saltator* share a common anatomical apparatus located in the antennae, the site of the time-compensated solar mechanism in the Monarch butterfly, *Danaus plexippus*. We show that the ability of sandhoppers to orientate using the sun is not perturbed by removal of, or unilaterally obscuring the antennae but, night-time compass orientation under moon-light is compromised. We also show that the antennae and brain exhibit rhythmic expression of core circadian genes that suggest these animals may have an antennal clock involved in lunar orientation.

The results obtained by Reppert and co-workers on the Monarch butterfly and our data presented here raise the question of whether, in arthropods, antennae are obligatory for time-compensated solar and/or lunar orientation. For the sun compass it appears that this is not the case since some lycosid spiders possess a sun compass but lack antennae^28^. Moreover, our tests show that *T. saltator* can orientate correctly using a sun compass after removal or unilateral occlusion of the antennae. These results are in contrast to *D. plexippus* which loses the ability to orientate effectively after these treatments.

The case for lunar orientation is somewhat similar; among the very few species of arthropods in which time-compensated lunar orientation has been demonstrated^9^,^28^,^29^,^30^ only the crustaceans *T. saltator* and *Tylos europaeus* possess antennae, whilst the spider *Arctosa variana* lack these appendages. In the ant *Formica rufa*^31^ and the earwig *Labidura riparia*^32^ the time compensation of the moon azimuth has not been definitively demonstrated, and while any general phylogenetic conclusions remain speculative, it appears that time-based moon orientation may be a feature of coastal crustaceans.

The existence of lunar and solar orientation mechanisms in the same organism is not without precedence; in *T. saltator* this has been demonstrated using behavioural experiments^13^. The existence of timing mechanisms synchronised with lunar phases and lunidian cycles was established many years ago^33^, but whether phenotypes occurring on monthly or circatidal (12.4 h) cycles were governed by dedicated non-circadian time-keepers remained unresolved. Recently however, evidence from behavioural, genetic and pharmacological experiments in the crustacean *Eurydice pulchra* and polychaete worm, *Platynereis dumerilii* has emerged supporting the notion that circatidal and circalunar phenotypes are orchestrated by timekeepers separate from the circadian clock^22^,^34^.

Given the well-documented role of clock genes, including *per* and *cry2* in transcriptional-translational regulatory feedback loops in other species, their function in Monarch butterfly time-compensation^3^ and temporal gating of olfactory function in insect antennae^35^,^36^,^37^, we postulate that *T. saltator* antennae also have a timing mechanism. In flies, light-entrained antennal clocks that govern olfactory sensitivity operate independent of the central oscillator and visual system^38^,^39^ and in Monarch butterflies ‘cryptochrome-centric’ clocks in the central brain and antennae play different roles in circadian timing and navigation^3^,^8^.

Our demonstration of daily oscillations in clock gene expression in the antennae of *T. saltator* reveals the existence of a putative molecular timekeeper in these tissues. Notwithstanding mounting evidence of the importance of post-transcriptional and post-translational events in the molecular clock^40^,^41^,^42^, and indeed rhythms in the absence of transcription^43^, it is a feature of many oscillatory systems that rhythmicity in clock gene expression reflects the functioning of the clock mechanism; in crustaceans free-running rhythms in core cock genes have been reported as evidence of a clock mechanism^22^,^44^. In the absence of available reagents and the apparent difficulty of measuring clock proteins in small crustaceans *in vivo*^22^, the clear daily fluctuations in *period* and *cryptochrome 2* are an indication that the antennae have the competence for time-keeping. This pattern of *Talcry2* expression in the antenna is reminiscent of antennal *cry2* in butterflies^3^ and although speculative at this stage, the antiphasic expression of *Talcry2* in the antennae and brain presents the tantalising prospect that, the phase differences in these discrete tissues reflect solar versus lunar timing function.

At present, we can’t rule out the possibility that the moon compass in *T. saltator* is not exclusive to the antennae. Nevertheless, our experiments show that whilst the antennae of *T. saltator* are not necessary for solar orientation, they are important in the functioning of a moon compass. Moreover, we demonstrate rhythmic expression of canonical clock gene transcripts in the brain and antennae, illuminating the possibility that the latter might have an important function in time-keeping, separate from that of daily locomotor rhythms. Taken together, our data suggest that the antennae accommodate a time-keeping component of the lunar compass, discrete from the solar clock, which is probably located in the brain or eye. Future work will aim to elucidate the cellular localization of these putative clocks in *T. saltator.*

## Additional Information

**How to cite this article**: Ugolini, A. *et al.* Evidence for discrete solar and lunar orientation mechanisms in the beach amphipod, *Talitrus saltator* Montagu (Crustacea, Amphipoda). *Sci. Rep.* **6**, 35575; doi: 10.1038/srep35575 (2016).

## Supplementary Material

Supplementary Information
