# Supplementary material for: Evidence for discrete solar and lunar orientation mechanisms in the beach amphipod, Talitrus saltator Montagu (Crustacea, Amphipoda)
Source: Sci Rep. 2016 Oct 19;6:35575. doi: 10.1038/srep35575 (PMC5069674; doi:10.1038/srep35575)
Supplement: Supplementary Information [file srep35575-s1.docx]

| **Method** | **Oligonucleotide name** | **Fluor/Sequence (5’-3’)** |
| --- | --- | --- |
|  |  |  |
| **qPCR standards synthesis** | TalAK STD F’ | TATTTGGGGGCACAATTTGA |
|  | TalAK STD R’ | T7-CCAAGGCCAGTTTTCTTGTC |
|  | Talclk STD F’ | TTCCTGTTCCTAGACCAGAGG |
|  | Talclk STD R’ | T7-ATTCTGGCTTGCTGTTCCAC |
|  | Talcry2 STD F’ | TGTTTGGAGGATGTGGACAA |
|  | Talcry2 STD R’ | T7-ATGACTTCGATGCCCATCTC |
|  | Talper STD F’ | CTCGAGATTCGGTGCATTTT |
|  | Talper STD R’ | T7-GCCATCACCTCTTCTTCCAA |
|  | Taltim STD F’ | TGCACTTGGCCGGCATA |
|  | Taltim STD R’ | T7-ACGGTTCTTCGGGATCTTTT |
|  |  |  |
| **Taqman™ assay primers and probes** | TalAKTaq F’ | TCGAGGAACGGCTGTCCTT |
|  | TalAKTaq R’ | AGCAGCGTCAACCATTTTGTT |
|  | TalAKTaq Probe | NED-CCTGAGCCAAGACAG |
|  |  |  |
|  | TalclkTaq F’ | TCGGTTACCTGCCATTCGA |
|  | TalclkTaq R’ | CAGGTCGTCCACATGGTAGTAATC |
|  | TalclkTaq Probe | FAM-TGCTAGGTACCTCAGGTTA |
|  |  |  |
|  | Talcry2Taq F’ | GCGCTCCCGCAGCTT |
|  | Talcry2Taq R’ | GGTCTTCCTCGAAACTCAGAACTG |
|  | Talcry2Taq Probe | FAM-TCAAGGAGTGGAATACG |
|  |  |  |
|  | TalperTaq F’ | GCCTCATGCCTCCATTCTACTG |
|  | TalperTaq R’ | CGCAGAGGCTTGGTAGCATT |
|  | TalperTaq Probe | VIC-CGAGTTCGCGAGAAC |
|  |  |  |
|  | TaltimTaq F’ | TGCACTTGGCCGGCATA |
|  | TaltimTaq R’ | CTCGTCCGTCGACGTAGACA |
|  | TaltimTaq Probe | VIC-CGGACCTGCTCTTG |

**Supplementary Methods**

Primer and Taqman MGB probe sequences used in generation of qPCR standards and qPCR assays of Talitrus saltator brain and antennae samples.

T7 phage promoter sequence 5’-TAATACGACTCACTATAGGGAGA-3’

**Quantitative PCR assays**

*Quantitative PCR Standards curves*

RNA standard curves were generated by *in vitro* transcription using T7phage promoter flanked PCR products (see Table above for primer sequences) as templates. PCRs were done using Bioline MyTaq Red mastermix in 3 x 50μl volumes with the following thermocycling profiles: 95°C for 5 mins activation, followed by 40 cycles of 95°C 15s, 55°C 45s and 72°C 1 min., followed by a 72°C 7 min. final extension. PCR products were pooled, purified using PCR clean-up kits (Bioline, UK) and eluted in 20μl DEPC-treated water. Products were then used for *in vitro* transcription using a T7 MegaShortScript kit (Ambion, Thermo Life Sciences, UK) according to the manufacturer’s instructions. Transcription products were purified on 10% 6M urea polyacrylamide gels, stained with ethidium bromide and products of expected size excised and eluted overnight in Probe Elution Buffer (Ambion, Thermo Life Sciences, UK) at RT. RNA was subsequently precipitated in three volumes of ethanol and resuspended in 30μ  water. RNA was quantified using a Nanodrop ND-1000 spectrophotometer (Thermo Life Sciences, UK) and copies of RNA calculated using Avogadro’s constant/moles. Standard curves in the range 10^10^ to 10^3^ copies were reverse transcribed using Tetro cDNA synthesis reagents (Bioline, UK) and random hexamer primers according to the manufacturer’s instructions.

*Sample RNA extraction*

Sample RNA from *T. saltator* heads (10 per time-point replicate) and antennae was extracted using Qiagen Universal RNA extraction columns following homogenization in Qiazol reagent. On-column DNAse digestion was performed to remove contaminating gDNA according to manufacturer’s instructions. 500ng total RNA was reverse transcribed as for standards (above) in 20ul reactions and then diluted 5-fold in DEPC-treated water.

*Quantitative PCR*

All qPCR reactions were done on an Applied Biosystems QuantStudio 12-Flex 384 well platform using Bioline Sensifast Probe II PCR mix and Taqman MGB probes (see Table above). Quantitative PCR reactions were done in 10μl reactions containing 2μl cDNA and with each target gene assayed in the same reaction as the reference gene *TalAK*. Each primer and probe was used at 0.25μl (2.5μM) per reaction. Cycling conditions were: 95°C 2min (activation) followed by 40 cycles of 95°C 10s, 60°C 15s. Data are expressed as copy number of target gene per copy of the reference gene.
